# Supplementary material for: SERCA Silencing Alleviates Aß(1-42)-Induced Toxicity in a C. elegans Model
Source: Int J Mol Sci. 2025 Sep 18;26(18):9126. doi: 10.3390/ijms26189126 (PMC12470765; doi:10.3390/ijms26189126)
Supplement: Supplementary file 1 [file ijms-26-09126-s001.zip › ijms-3823379-supplementary.pdf]

Supplementary Figures

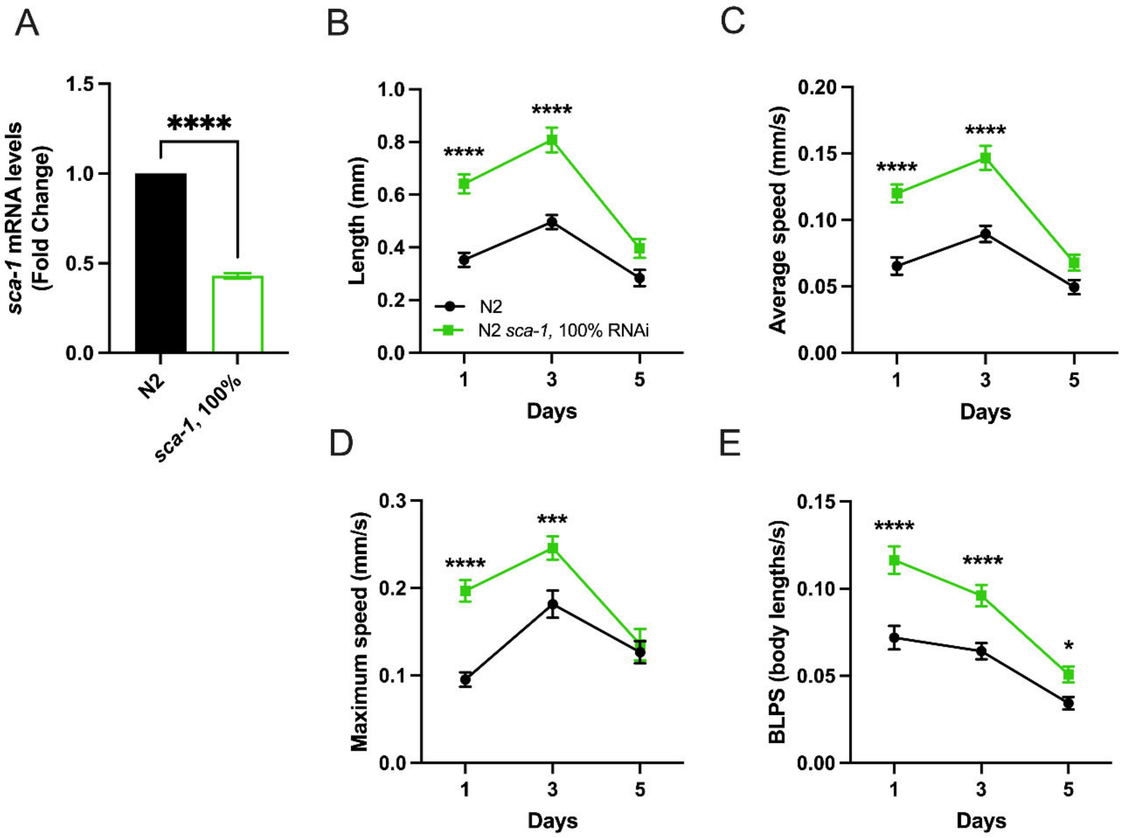

Supplementary Figure S1. Impact of *sca-1* silencing on N2 worms in free mobility assays at day 1, 3 and 5 of adulthood. A) Effect of *sca-1* RNAi on *sca-1* mRNA levels in wild type worms, N2 (n = 3, experiments). Panels B to E show the following mobility parameters of N2 fed with control RNAi (L4440) and 100% *sca-1* RNAi at day 1, 3 and 5 of adulthood: B) Length C) Average speed D) Maximum speed E) BLPS (body lengths per second). Results represent three biological replicates (n ≅ 60 worms/condition). Data are presented as mean ± s.e.m. * $p < 0.05$; ** $p < 0.01$; *** $p < 0.001$; **** $p < 0.0001$.

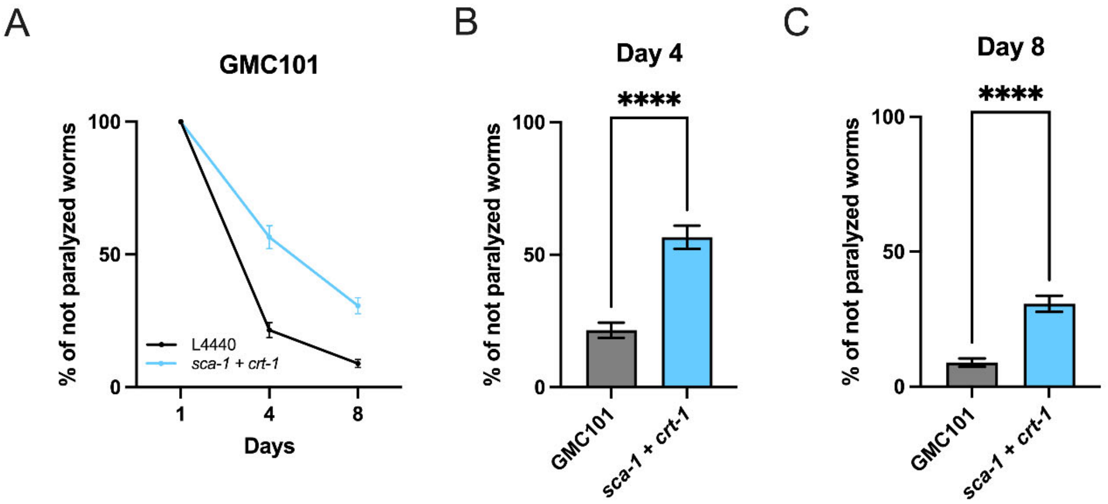

D

% of not paralyzed worms

	Day 4	Day 8
***sca-1*, 100% RNAi**	22.6	42.3
***crt-1*, 100% RNAi**	14.9	42.8
**(*sca-1* + *crt-1*), 100% RNAi**	35.1	21.8

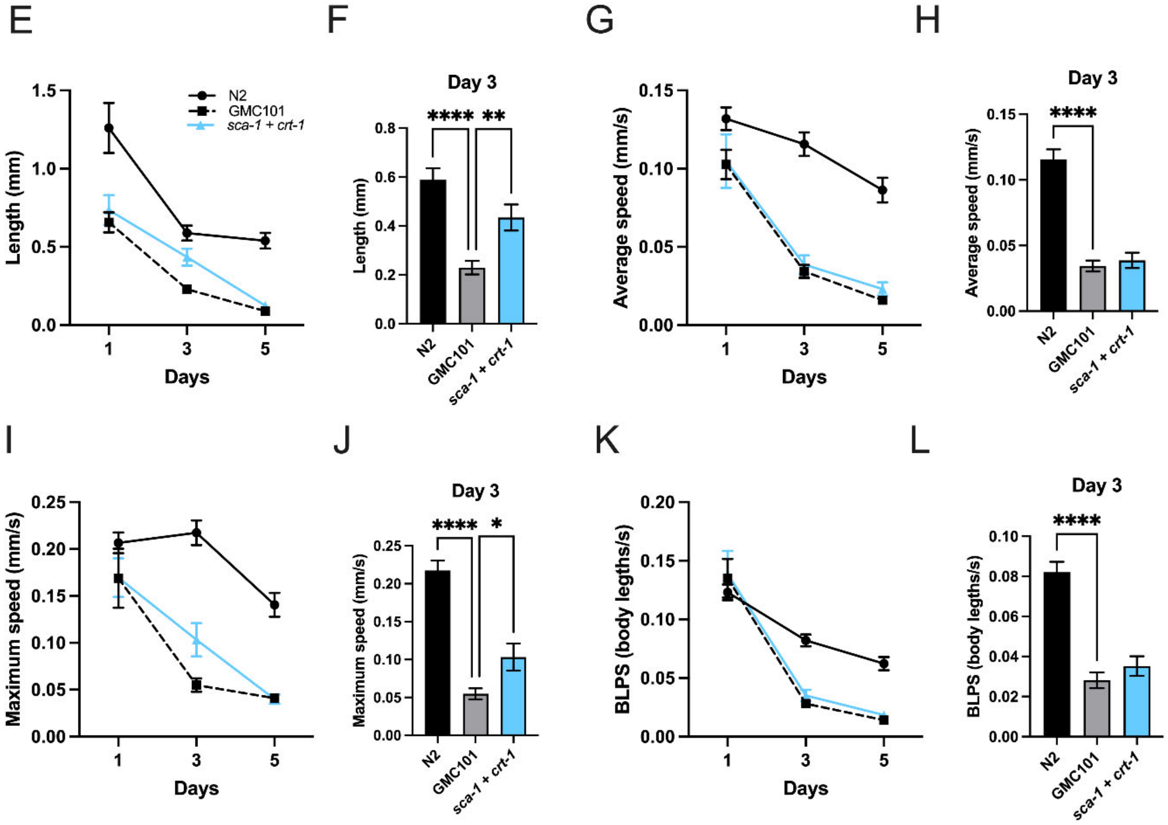

Supplementary Figure S2. Impact of additive *crt-1* and *sca-1* silencing on temperature-induced paralysis and mobility of GMC101 worms. A) Percentage of non-paralyzed GMC101 worms fed with control RNAi (L4440) and a mixture of *crt-1* + *sca-1* RNAi, over

time. B-C) Percentage of non-paralyzed GMC101 worms at day 4 (B), and at day 8 (C). D) Table summarizing the percentage of non-paralyzed GMC101 worms at day 4 and day 8 for the knockdown of *sca-1*, *crt-1*, or their combination regarding fed animals with control bacteria (L4440). Panels E, G, I and K show the following mobility parameters of N2 and GMC101 fed with control RNAi (L4440) or *crt-1* + *sca-1* RNAi, at day 1, 3 and 5 of adulthood: E) Length, G) Average speed, I) Maximum speed, and K) BLPS (body lengths per second). The following mobility parameters are represented at day 3 of adulthood: F) Length, H) Average speed, J) Maximum speed, and L) BLPS (body lengths per second). For paralysis and mobility assays, results represent three biological replicates ($n \cong 300$ worms/condition and $n \cong 60$ worms/condition, respectively). Data are presented as mean ± s.e.m. * $p < 0.05$; ** $p < 0.01$; *** $p < 0.001$; **** $p < 0.0001$.

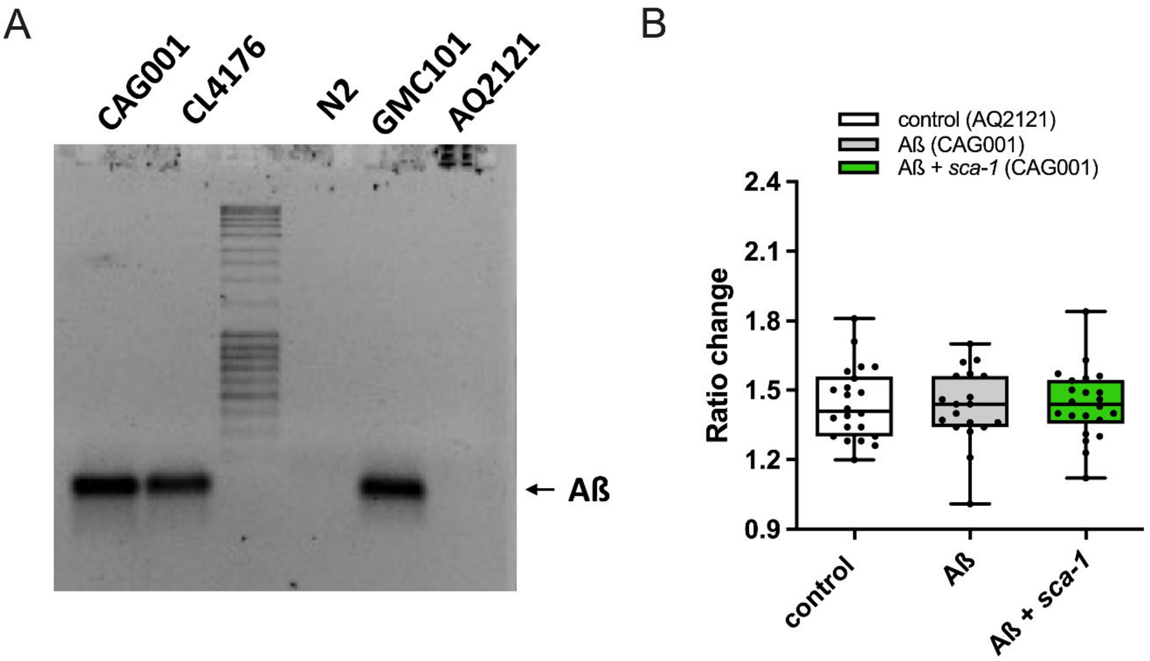

Supplementary Figure S3. A) PCR of genotype Aß verification of new CAG001 strain originated from the crossing of CL4176 (Aß overexpressing strain) with AQ2121 (body wall muscle cytosolic ratiometric Ca^{2+} sensor strain). Wild type strain, N2, is used as Aß negative control and GMC101 (Aß overexpressing strain) as Aß positive control. B) Effect of $A\beta_{(1\text{-}42)}$ overexpression and *sca-1* silencing on basal FRET ratios of vulva muscles. All experiments are the result of three biological replicates, each containing almost 3 technical replicates (n = 10 – 22). Data are mean ± s.e.m. * $p < 0.05$; ** $p < 0.01$; *** $p < 0.001$; **** $p < 0.0001$.
